# TIGAR cooperated with glycolysis to inhibit the apoptosis of leukemia cells and associated with poor prognosis in patients with cytogenetically normal acute myeloid leukemia

**DOI:** 10.1186/s13045-016-0360-4

**Published:** 2016-11-25

**Authors:** Sixuan Qian, Jianyong Li, Ming Hong, Yu Zhu, Huihui Zhao, Yue Xie, Jiayu Huang, Yun Lian, Yanru Li, Shuai Wang, Jianping Mao, Yaoyu Chen

**Affiliations:** Department of Hematology, The First Affiliated Hospital of Nanjing Medical University, Jiangsu Province Hospital, 300 Guangzhou Road, Nanjing, 210029 China

**Keywords:** *TIGAR*, Glycolysis, Acute myeloid leukemia, Apoptosis, Survival

## Abstract

**Background:**

Cancer cells show increased glycolysis and take advantage of this metabolic pathway to generate ATP. The TP53-induced glycolysis and apoptosis regulator (TIGAR) inhibits aerobic glycolysis and protects tumor cells from intracellular reactive oxygen species (ROS)-associated apoptosis. However, the function of TIGAR in glycolysis and survival of acute myeloid leukemia cells remains unclear.

**Methods:**

We analyzed TIGAR expression in cytogenetically normal (CN-) AML patients and the correlations with clinical and biological parameters. In vivo and in vitro, we tested whether glycolysis may induce TIGAR expression and evaluated the combination effect of glycolysis inhibitor and *TIGAR* knockdown on human leukemia cell proliferation.

**Results:**

High TIGAR expression was an independent predictor of poor survival and high incidence of relapse in adult patients with CN-AML. TIGAR also showed high expression in multiple human leukemia cell lines and knockdown of *TIGAR* activated glycolysis through PFKFB3 upregulation in human leukemia cells. Knockdown of *TIGAR* inhibited the proliferation of human leukemia cells and sensitized leukemia cells to glycolysis inhibitor both in vitro and in vivo. Furthermore, *TIGAR* knockdown in combination with glycolysis inhibitor 2-DG led leukemia cells to apoptosis. In addition, the p53 activator Nutlin-3α showed a significant combinational effect with *TIGAR* knockdown in leukemia cells. However, TIGAR expression and its anti-apoptotic effects were uncoupled from overexpression of exogenous p53 in leukemia cells.

**Conclusions:**

TIGAR might be a predictor of poor survival and high incidence of relapse in AML patients, and the combination of TIGAR inhibitors with anti-glycolytic agents may be novel therapies for the future clinical use in AML patients.

**Electronic supplementary material:**

The online version of this article (doi:10.1186/s13045-016-0360-4) contains supplementary material, which is available to authorized users.

## Background

“Warburg effect” is a fundamental metabolic change during malignant transformation in human cancer [1–3]. Under this condition, most cancer cells predominantly produced energy by a high rate of glycolysis and showed an elevated fructose-2, 6-bisphosphate (Fru-2,6-P2) levels [1, 2]. These metabolic pathways underpinning the abnormal growth, proliferation, and survival of cancer cells were modulated by a couple of glycolytic enzymes [4, 5]. As well as solid tumors, human leukemia cells also exhibited the increased rating of aerobic glycolysis and generated ATP as the main energy source [5, 6].

Many oncogenes and tumor suppressors regulated the expression of glycolytic enzymes [7]. TIGAR, a p53-inducible glycolysis and apoptosis regulator, has a functional sequence similar to the bisphosphatase domain (FBPase-2) of 6-phosphofructo-2-kinase (PFK-2/FBPase) [8]. The functions of TIGAR were potentially relevant to cancer initiation and progression [8]. On the one hand, high expression of TIGAR in human cancer may protect cancer cells from cell death [9]. On the other hand, TIGAR inhibited glycolysis through Fru-2,6-P2 degradation, directing metabolism into the pentose phosphate pathway (PPP) to produce NADPH and glutathione (GSH) as anti-oxidants, and ribose-5-phosphate for nucleotide synthesis [10]. TIGAR also showed high expression among several cancer types, including human colon tumors [4], breast cancer [11, 12], and glioblastoma [13–15], which suggesting that upregulated TIGAR expression may support, rather than inhibit, cancer development [1]. High TIGAR expression correlated with the increased tumor survival/burden, while TIGAR depletion promoted the apoptosis rate of cancer cells [12, 16–18]. TIGAR depletion also enhanced the epirubicin-induced activation of autophagy [19]. In addition, knockdown of *TIGAR* gene increased Fru-2,6-P2 and reactive oxygen species (ROS) levels and decreased GSH levels in glioblastoma cells [14].

However, the function of TIGAR in human chronic or acute leukemia remains unknown. In this study, we showed that the expression of TIGAR in patients with cytogenetically normal acute myeloid leukemia (CN-AML) correlated with the clinical features and outcomes. The high TIGAR expression in AML might be an independent prognostic factor for survival in patients with CN-AML. Knockdown of *TIGAR* inhibited the proliferation of human leukemia cells and sensitized leukemia cells to glycolysis inhibitor 2-deoxy-d-glucose (2-DG) both in vitro and in vivo, which may be due to increased apoptosis rate of leukemia cells. Our results suggested that TIGAR might be a predictor of poor survival and a novel therapeutic target for human AML.

## Methods

### Patients and samples

One hundred sixteen patients, aged ≥14 years, with previously untreated CN-AML attended this study. All patients were diagnosed for AML. All those patients had complete clinical data available, and enough cryopreserved bone marrow (BM) samples taken at diagnosis, for analysis. Twenty health donors attended the study as the control. Among 116 patients, 109 patients were treated and followed up (until death or for a period of up to 53 months, between October 2007 and February 2013) at the Hematology Department of the First Affiliated Hospital of Nanjing Medical University (Nanjing, People’s Republic of China). All 109 patients received cytarabine-based intensive induction and consolidation chemotherapy. This study was approved by the institutional review board of the First Affiliated Hospital of Nanjing Medical University and carried out in accordance with the Declaration of Helsinki. All patients and normal donors provided written informed consent for this study.

### Cytogenetic and mutation analyses

BM cells were harvested directly or after 1–3 days of unstimulated culture, as described previously [1]. Metaphase cells were banded via an improved heat treatment and Giemsa R-banding method. The diagnosis of a normal karyotype was based on conventional cytogenetic examination of at least 20 metaphases. Genomic DNA was isolated from BM specimens. Mutation analysis of five relevant molecular marker genes (NPM1, CEBPA, FLT3-ITD, KIT, and p53) was carried out as described previously [20, 21].

### Outcome measures

The primary endpoints were overall survival (OS; duration from diagnosis to death from any cause), disease-free survival (DFS; time from achievement of complete remission (CR) until relapse or death), and morphologic leukemia relapse (hematologic and/or extramedullary). For analyses of DFS, failure was considered to be clinical or hematologic relapse or death from any cause; patients alive and in CR were censored at last follow-up. For analyses of OS, failure was considered to be death from any cause; patients alive were censored at the date of last contact.

### Western blot

Cells were lysed in RIPA buffer containing Halt Protease and Phosphatase Inhibitor Mixture (Thermo Scientific). Lysates were spun at 16,000×*g* at 4 °C for 30 min and normalized for protein concentration. Western blotting was performed as follows: total tumor lysates were separated by SDS/PAGE and electrotransferred to nitrocellulose membrane (Invitrogen). Membranes were blocked in PBS and 0.1% (*vol*/*vol*) Tween-20 (PBS-T) and 4% (*wt*/*vol*) nonfat dry milk (Bio-Rad) for 1 h on a shaker at room temperature. Primary antibodies were added to the blocking solution at 1:500 (TIGAR; Abcam, 37910), 1:500 (GSH; Abcam, 19534), 1:500 (PFKFB3; Abcam, 96699), and 1:1000 (Actin; Abcam, 3280) dilutions and incubated overnight and a rocker at 4 °C. Immunoblottings were washed three times, 5 min each with PBS-T, and secondary antibody was added at 1:10,000 dilution into PBS-T milk for 1 h on a shaker at room temperature. After several washes, enhanced chemiluminescence (ECL) reactions were performed according to the manufacturer’s recommendations (SuperSignal West Dura Extended Duration Substrate; Thermo Scientific).

### Quantitative real-time reverse transcription PCR

The relative *TIGAR* mRNA expression was determined by comparing the *TIGAR* expression relative to GAPDH. The *TIGAR* expression was compared among other 116 AML patients by using the real-time quantitative PCR and the 2^−ΔΔCt^ method. The ΔCt of health donor was used as a control value for each AML patient. Patients with *TIGAR* expression values above the median of all patients were defined as having high *TIGAR* expression (*TIGAR* ^high^), while all other patients were considered to have low *TIGAR* expression (*TIGAR* ^low^).

### Cell lines

HL-60, K562, Jurkat, and NB-4 cells (ATCC, USA) were cultured in RPMI1640 (GIBCO, USA), 10% fetal bovine serum, 2 mM l-glutamine, 50 U/ml penicillin, and 50 μg/ml streptomycin. All these cell lines were authenticated and tested for mycoplasma contamination. Cells were treated with 400 μM cobalt chloride (CoCl_2_) (Amresco, USA) for 48 h to induce glycolysis, or with 1 mg/ml 2-deoxy-d-glucose (2-DG) (Sigma-Aldrich, USA) for 48 h to suppress glycolysis.

### Short hairpin RNA and gene overexpression constructs

To inhibit *TIGAR* mRNA expression, small interfering RNAs (siRNA) matching nucleotide region 565–583 (TTAGCAGCCAGTGTCTTAG, TIGAR siRNA) of the human *TIGAR* cDNA sequence were synthesized as an antisense, and a scramble sequence (TTACCGAGACCGTACGTAT) was synthesized as a control. The *TIGAR* and scramble sequence were further cloned into the pSRL-SIH1-H1-Puro lentivirus vector. *TIGAR* and p53 cDNA was ordered and cloned into pcDNA3 vector.

### Lentivirus and infection

Lentiviral supernatants were generated according to the established protocol. A medium was replaced and after 24 h. The scramble shRNA or *TIGAR* shRNA lentivirus transduced leukemia cells were selected by puromycin for 48 h and used.

### Measurement of apoptosis and cell death

HL-60 cells and NB-4 with scramble or shRNA-*TIGA*R were treated with CoCl_2_ or 2-DG for 48 h. After treatment, aliquots were removed and counted by trypan blue (Sigma-Aldrich) exclusion in duplicate. Apoptosis was quantified by phosphatidylserine externalization. Briefly, the samples were stained with Annexin V-FITC and propidium iodide (PI) or 7AAD according to the manufacturer’s recommendations. Flow cytometry (FACS Calibur; BD Biosciences) enabled the distinction of viable cells (Annexin V-FITC^−^, PI^−^) from those in apoptosis (Annexin V-FITC^+^, PI^−^) [22]. Annexin V-FITC^−^, PI^+^ population was defined as dead/necrosis cells. Except where documented, all results were shown as a mean plus or minus SD.

### Measurement of intracellular ROS

Human leukemia cells in 24-well plates were incubated at 37 °C for 30 min with 500 μl of 10 μmol/L DCFH-DA probe (S0033; Beyotime Institute of Biotechnology, Haimen, China), with shaking every 5 min. The cells were then washed with PBS (three times, 5 min each) to remove any remaining extracellular DCFH-DA probe [23]. The fluorescence intensity, representing cellular ROS levels, was detected using a Gemini XPS fluorimetric microplate reader (MolecularDevices, Shanghai, China), with excitation and emission wavelengths of 488 and 525 nm, respectively.

### Measurement of intracellular F2,6BP

HL-60 cells were centrifuged at 200×*g*, resuspended in 20 volumes of 0.05 N NaOH and then one volume of 0.1 N NaOH to obtain a pH >11, vortexed for 10 s, incubated at 80 °C for 5 min and cooled in an ice bath. Cell extracts were neutralized to pH 7.2 with ice-cold acetic acid in the presence of 20 mM HEPES. Samples were incubated at 25 °C for 2 min in the following assay mixture: 50 mM Tris, 2 mM Mg^2+^, 1 mM F6P, 0.15 mM NAD, 10 u/l PPi-dependent PFK1, 0.45 kU/l aldolase, 5 kU/l triosephosphate isomerase, and 1.7 kU/l glycerol-3-phoshate dehydrogenase (Sigma). 0.5 mM pyrophosphate was added and the rate of change in absorbance (OD = 339 nm) per min was followed for 5 min. F2,6BP was calculated based on a calibration curve produced by measuring 0.1 to 1 pmol of F2,6BP (Sigma) and normalized to total cellular protein.

### Tumor xenografts

Mice were maintained and handled in accordance with Nanjing Medical University Animal Care and Use Committee protocols and regulations. HL-60 cells with scramble shRNA or shRNA against TIGAR were cultured in RPMI1640 supplemented with 10% FBS. BALB/c (nu/nu) nude female mice (6–8 weeks old, *n* = 10) were inoculated with 1 × 10^6^ cells through i.p. injection. The human leukemia cells were measured by FACS. Drug treatment started 7 days after implant. Those animals were assigned randomly to different groups. Animals received vehicle (5% dextrose, 10 ml/kg, orally, once a day) or 2-DG (2 g/kg, orally, once a day) for the duration of the study. Data were shown as mean ± SD, and differences are considered statistically significant at *p* < 0.05 by Student’s *t* test.

### Statistical analyses

Data were analyzed using SPSS version 16.0 (IBM, USA). Statistical significance was considered at *P* < 0.05. Possible differences between continuous variables were analyzed using Student’s *t* test. Data are represented as mean with SD as error bars unless otherwise mentioned. No power analysis was used to pre-determine sample size. Chi-square or Fisher’s exact tests were performed to compare incidences. The Kaplan-Meier method was employed to estimate survival probabilities, and the log-rank test for univariate comparisons. The probabilities of relapse were calculated by cumulative incidence curves. The associations between TIGAR expression or other characteristics and OS were studied using a Cox’s proportional hazards regression model.

## Results

### TIGAR upregulation is associated with poor prognosis in AML patients

**Fig. 1:**
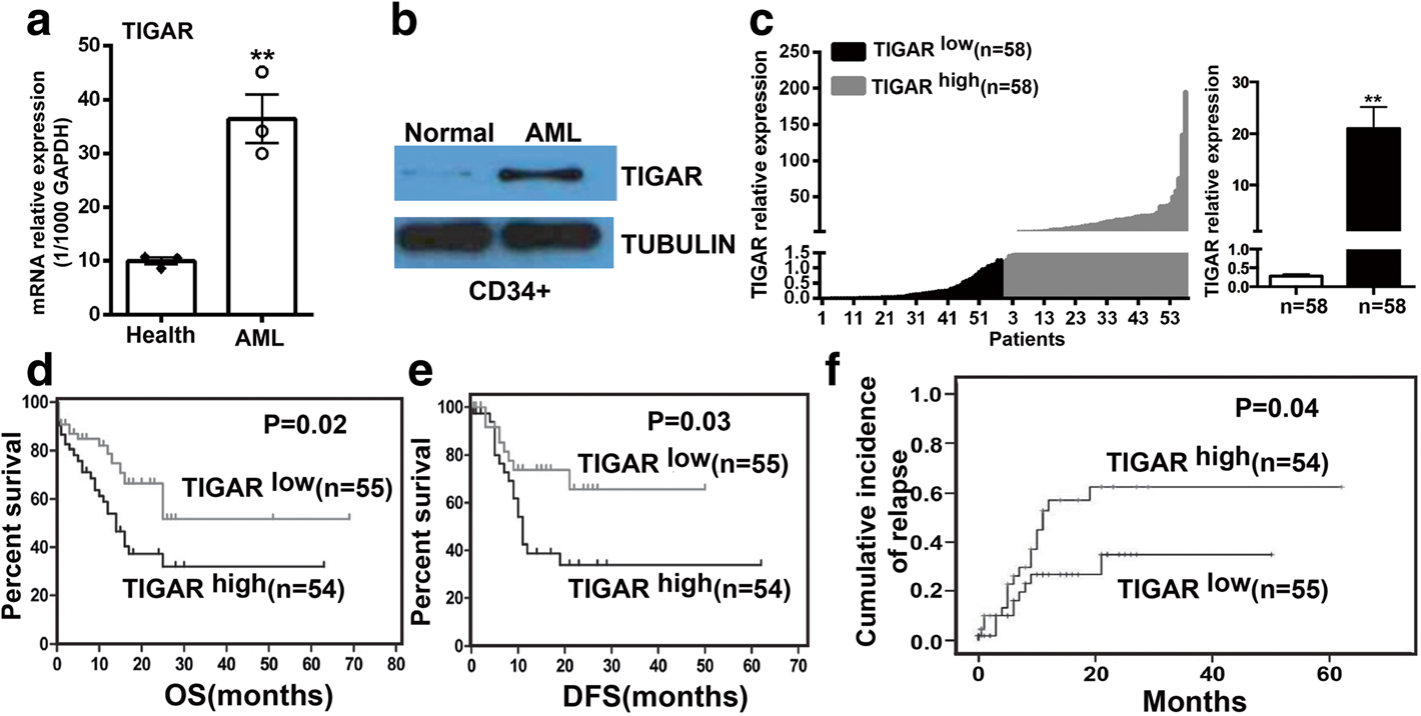
TIGAR upregulation was associated with poor prognosis in AML patients. **a** Real-time PCR showed that *TIGAR* mRNA was significantly upregulated in PB cells from three AML patients in comparison with three healthy donors. The *TIGAR* expression was normalized to 1000 copies of GAPDH expression. **b** Western blotting analysis showed that the protein expression of TIGAR protein was increased in CD34^+^ BM cells from AML patient versus healthy donor. **c** Real-time PCR showed that the relative expression of TIGAR mRNA between *TIGAR* ^low^ and *TIGAR* ^high^ patients. The result represented as mean with S.E. as error bars. **d**–**f** Patients from *TIGAR* ^low^ group presented significantly longer overall survival (OS) (*P* = 0.021) (**d**) and disease-free survival (DFS) (*P* = 0.028) (**e**) and lower cumulative incidence of relapse (*P* = 0.044) (**f**) than patients from *TIGAR* ^high^ group

The expression of TIGAR was evaluated in healthy donor and primary AML samples by real-time PCR. TIGAR was significantly upregulated in primary AML blood cells in comparison with healthy human blood cells (Fig. 1a). The upregulation of TIGAR was also shown in CD34^+^ BM cells from healthy donor or AML patients by western blotting (Fig. 1b). To understand the expression of TIGAR in the population of AML patients, we collected 116 AML patients and measured the TIGAR expression in BM cells from AML patients by real-time PCR. Those patients from *TIGAR* ^high^ group showed a robust upregulation of *TIGAR* gene in BM cells in comparison with patients from *TIGAR* ^low^ group (Fig. 1c).

To investigate whether upregulation of TIGAR was associated with prognosis in AML patients, we further analyzed the association of *TIGAR* expression with prognosis in those AML patients with median age 48 years (range, 12–86 years). Eighty-two (70.7%) patients were aged <60 years (“younger patients”), and 34 (29.3%) patients were aged ≥60 years (“older patients”). The clinical characteristics of these patients were shown in Table 1. There was no significant difference between the two groups for most clinical characteristics, including white blood cell count, hemoglobin level, platelet count, % peripheral blood (PB) blasts, and % BM blasts. In addition, no association was found between *TIGAR* expression and mutations in the *NPM1*, *FLT3-ITD*, *c-KIT*, or *P53* genes; however, the patients from *TIGAR* ^low^ group were more prone to have high *CEBPA* expression (*P* = 0.0453). The median survival of the entire cohort was 25 months (2–69 months). The 109 patients from *TIGAR* ^high^ and *TIGAR* ^low^ groups had a similar rate of complete response (CR) (74.5 vs. 72.7%). However, the 55 patients from *TIGAR* ^low^ group showed a significantly longer OS (*P* = 0.021) (Fig. 1d) and disease-free survival (DFS) (*P* = 0.028) (Fig. 1e) and a lower cumulative incidence of relapse (*P* = 0.044) (Fig. 1f) than patients from *TIGAR* ^high^ group. The 54 patients from *TIGAR* ^high^ group also showed a trend towards a higher relapse rate than those from *TIGAR* ^low^ group (29.1 vs. 18.2%), although statistical significance was not reached.

**Table 1:** Clinical characteristics of the patients with CN-AML according to their TIGAR expression levels

	TIGAR^high^	TIGAR^low^	*P*
Age (years), median (range)	49 (15–80)	47 (12–86)	0.798
Gender, male/female	29/29	28/30	1.000
WBC, median (range) (×10^9^/L)	26 (0.6–291)	34 (1.3–299)	0.995
Hb, median (range) (g/L)	79 (39–154)	87 (39–148)	0.398
PLT, median (range) (×10^9^/L)	42 (10–190)	37 (2–295)	0.190
PB blasts (%), median (range)	66 (0–98)	65 (0–96)	0.525
BM blasts (%), median (range)	75 (11.6–96.2)	72 (24–93.6)	0.870
NPM1 (+)	25.0% (12/48)	22.0% (11/50)	0.726
CEBPA (+)	14.6% (7/48)	31.9% (15/47)	0.045
FLT3-ITD (+)	12.5% (6/48)	15.7% (8/51)	0.649
C-KIT (+)	6.3 (3/48)	6.3% (3/48)	1.000
p53 mutation	2.1% (1/48)	2.0%(1/51)	1.000

*BM* bone marrow, *Hb* hemoglobin, *PB* peripheral blood, *PLT* platelets, *WBC* white blood cells

A multivariate analysis was conducted to determine the prognostic significance of *TIGAR* expression with consideration of other known risk factors, including age, white blood cell count (WBC), and different chemotherapy regimens (Table 2). We found that low *TIGAR* expression was associated with a reduction in the risk of death (*P* = 0.023; Table 3). Younger age was also associated with longer survival (*P* = 0.025, Table 3). In addition, a high proportion of BM blasts (*P* = 0.058, Table 3) and chemotherapy (*P* = 0.078, Table 3) may be also involved into longer survival of AML patients.

**Table 2:** Chemotherapy regimens of AML patients

Induction	Consolidation	Case numbers	Dosage of anthracyclines (each course)
IA	Intermediate dose cytarabine	74	Idarubicin 12 mg/m^2^/day, IV, day 1 to 3
CAG	CAG	12	Aclarubicin 10 mg/day, IV, day 1 to 8
DCAG	DCAG	23	Aclarubicin 10 mg/day, IV, day 3 to 6

IA: idarubucin 12 mg/m^2^ once daily intravenous (IV) from day 1 to 3 combined with cytarabine 100 mg/m^2^ continuous intravenous (CIV) from day 1 to 7. CAG: granulocyte colony-stimulating factor (G-CSF) of 300 μg/day (day 0–14) subcutaneous injection (SQ) for priming combined with cytarabine of 10 mg/m^2^ SQ q12h for 14 days (day 1–14), aclarubicin of 10 mg/day IV for 8 days (day 1–8). The G-CSF priming was discontinued if white blood count (WBC) was >20 × 10^9^/L. DCAG: decitabine of 15 mg/m^2^ IV for 5 days (day 1–5) and G-CSF of 300 μg/day (day 0–9) SQ for priming combined with cytarabine of 10 mg/m^2^ SQ q12h for 7 days (day 3–9), aclarubicin of 10 mg/day IV for 4 days (day 3–6). The G-CSF priming was discontinued if WBC was >20 × 10^9^/L

**Table 3:** Multivariate analysis of factors associated with OS in patients with CN-AML

	*B*	SE	Wald	*P*	Exp (B)	95% CI
Age	0.039	0.017	5.017	*0.025*	1.040	1.005 to 1.076
WBC	−0.004	0.004	1.250	0.264	0.996	0.989 to 1.003
Hb	0.008	0.009	0.769	0.381	1.008	0.990 to 1.027
PLT	0.003	0.003	1.242	0.265	1.003	0.997 to 1.009
PB blasts	−0.006	0.011	0.326	0.568	0.994	0.972 to 1.016
BM blasts	0.029	0.015	3.584	0.058	1.030	0.999 to 1.061
Allo-SCT	0.323	0.614	0.277	0.599	0.724	0.217 to 2.411
NPM1	−0.197	0.432	0.209	0.648	0.821	0.352 to 1.914
CEBPA	−0.880	0.543	2.620	0.106	0.415	0.143 to 1.204
FLT3-ITD	−0.980	0.804	1.484	0.223	0.375	0.078 to 1.816
C-KIT	−12.589	644.378	0.000	0.984	0.000	
TIGAR	0.868	0.381	5.200	*0.023*	2.383	1.130 to 5.025
Chemotherapy	0.587	0.333	3.114	0.078	1.799	0.937 to 3.455

*Allo-SCT* allogeneic hematopoietic stem cell transplantation, *BM* bone marrow, *Hb* hemoglobin, *PB* peripheral blood, *PLT* platelets, *WBC* white blood cells

The italicized number represented *P* < 0.05

### TIGAR showed a high expression in human leukemia cell lines and glycolysis induced the expression of TIGAR

**Fig. 2:**
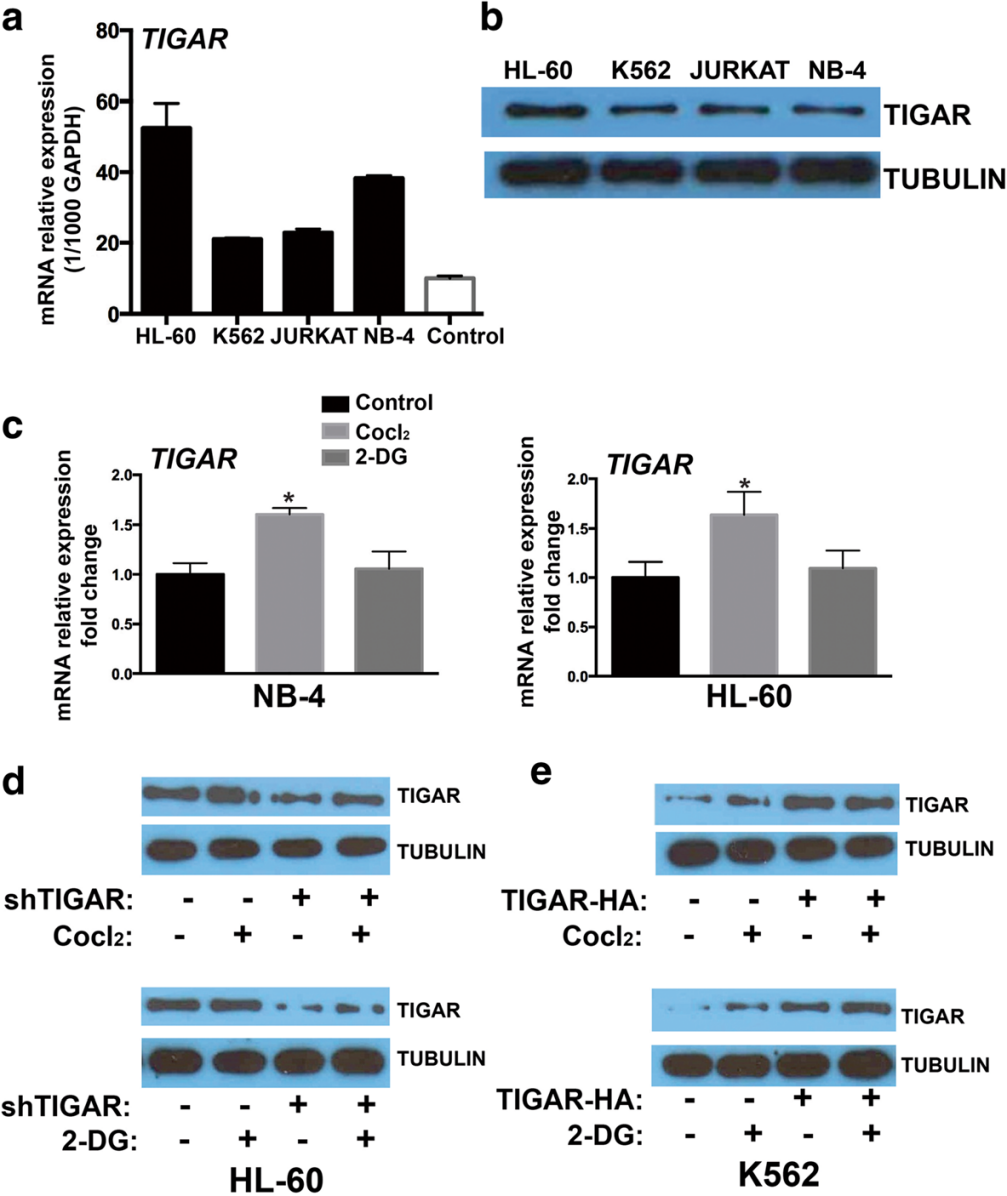
TIGAR showed a high expression in human leukemia cell lines, and glycolysis induced the expression of TIGAR. **a**, **b** TIGAR mRNA and protein was compared among multiple leukemia cell lines and normal cells. **c** *TIGAR* mRNA level was compared by real time PCR in both HL-60 and NB-4 cells treated with or without CoCl_2_ and 2-DG. **d** Western blotting showed the expression of TIGAR in HL-60 cells with or without *TIGAR* knockdown. **e** Western blotting showed the expression of TIGAR in K562 cells with or without *TIGAR* overexpression

Because p53 null or mutant human tumor cell lines showed a significant high basal level of TIGAR protein expression regulated by p53-independent mechanisms [8], we decided to test the expression of TIGAR in several established human p53 null or mutant acute leukemia cell lines to identify the leukemia cell line with high expression of TIGAR. Four different acute leukemia cell lines: HL-60, K562, Jurkat, and NB-4 were tested. HL-60 and Jurkat were p53 null leukemia cell lines while K562 and NB-4 were p53-mutant leukemia cell lines. Among them, HL-60 and NB-4 were acute promyelocytic leukemia cell lines (the M3 subtype of AML). The K562 was derived from a CML patient in blast crisis. Jurkat was acute lymphoblastic leukemia cell line. Consistent with the previous study in human tumor cell lines, TIGAR was highly expressed in those p53 null or mutant leukemia cell lines than in normal cells, particularly for HL-60 cells (Fig. 2a, b). Therefore, HL-60 and NB-4 acute promyelocytic leukemia cell lines were selected for subsequent in vitro or in vivo experiments. The K562 with a relative low expression of TIGAR was also tested.

Next, we tested whether glycolysis may induce TIGAR expression in human acute leukemia cells. CoCl_2_ was used to stimulate glycolysis, and the glycolytic inhibitor 2-DG was used to block the glycolysis in leukemia cells [24, 25]. We showed that CoCl_2_ increased the F2,6BP while 2-DG reduced the F2,6BP in HL-60 cells (Additional file 1: Figure S1). The effects of CoCl_2_ and 2-DG on the expression of TIGAR were also tested in HL-60 and NB-4 cells. CoCl_2_ significantly increased mRNA expression of *TIGAR* in both HL-60 and NB-4 cells (Fig. 2c). In contrast, the glycolytic inhibitor 2-DG did not affect the expression of *TIGAR* in leukemia cells (Fig. 2c). In addition, we validated that the expression of TIGAR was induced by CoCl_2_ but not 2-DG, and the CoCl_2_-induced expression of TIGAR was reversed by *TIGAR* knockdown in HL-60 cells (Fig. 2d). We also overexpressed *TIGAR* in K562 cells (*TIGAR* low expressed acute leukemia cell line) and found that 2-DG but not CoCl_2_ induced the expression of TIGAR in K562 cells slightly (Fig. 2e). Neither 2-DG nor CoCl_2_ further increased the expression of TIGAR in *TIGAR*-overexpressed K562 cells (Fig. 2e). Those results suggested that some human acute leukemia cells showed a high expression of TIGAR, and glycolysis may induce the TIGAR expression in human leukemia cells.

### TIGAR regulated the glycolysis through PFKFB3 in human acute leukemia cells

**Fig. 3:**
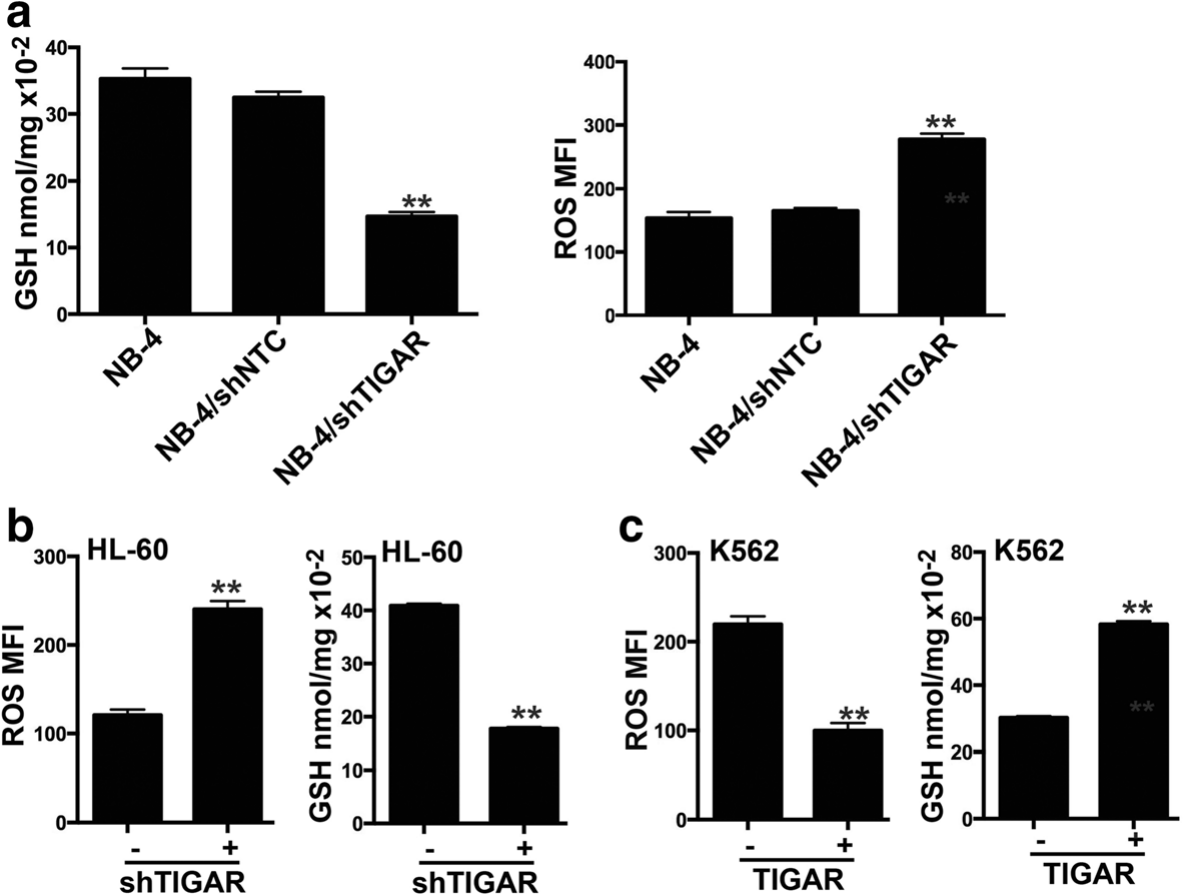
TIGAR regulated the glycolysis through PFKFB3 in leukemia cells. **a**, **b** *TIGAR* knockdown reduced the GSH level and increased the ROS level in NB-4 cells (**a**) and HL-60 cells (**b**). **c** *TIGAR* overexpression increased the GSH level and reduced the ROS level in K562 cells

Next, we investigated whether TIGAR regulated the glycolysis in leukemia cells. As most malignant cells were highly glycolytic and produced high levels of ROS and showed low levels of GSH, we first tested the effect of *TIGAR* knockdown on ROS and GSH in leukemia cells. We showed that knockdown of *TIGAR* reduced the GSH level and increased the ROS level in NB-4 cells (Fig. 3a). The similar results were also observed in HL-60 cells (Fig. 3b). In contrast, we found that overexpression of *TIGAR* increased the GSH level and reduced the ROS level in K562 cells (Fig. 3c). The potential mechanisms of TIGAR regulating the glycolysis were also tested in leukemia cells. PFKFB3 was an important glycolytic activator and active PFKFB3 induced PFK1 activity and led to glycolysis in cancer cells (Additional file 2: Figure S2a). Therefore, we determined to test whether TIGAR affected the expression of PFKFB in leukemia cells. Knockdown of *TIGAR* robustly increased the expression of PFKFB3 in HL-60 cells while overexpression of *TIGAR* reduced the expression of PFKFB3 in K562 cells (Additional file 2: Figure S2b). Furthermore, we found that the AML drug decitabine, hypomethylating DNA by inhibiting DNA methyltransferase, significantly reduced the expression of TIGAR while induced the expression of PFKFB3 in HL-60 cells (Additional file 2: Figure S2c). In addition, Cocl_2_ induced the TIGAR and reduced PFKFB3 while 2-DG induced PFKFB3 in decitabine treated HL-60 cells (Additional file 2: Figure S2c). Similarly, Cocl_2_ induced TIGAR and reduced PFKFB3 in NB-4 cells (Additional file 2: Figure S2d). Those results suggested that PFKFB3 might also be a potential mechanism of TIGAR regulating glycolysis in human leukemia cells.

### *TIGAR* knockdown inhibited the proliferation of leukemia cells and sensitized leukemia cells to glycolysis inhibition in vitro

**Fig. 4:**
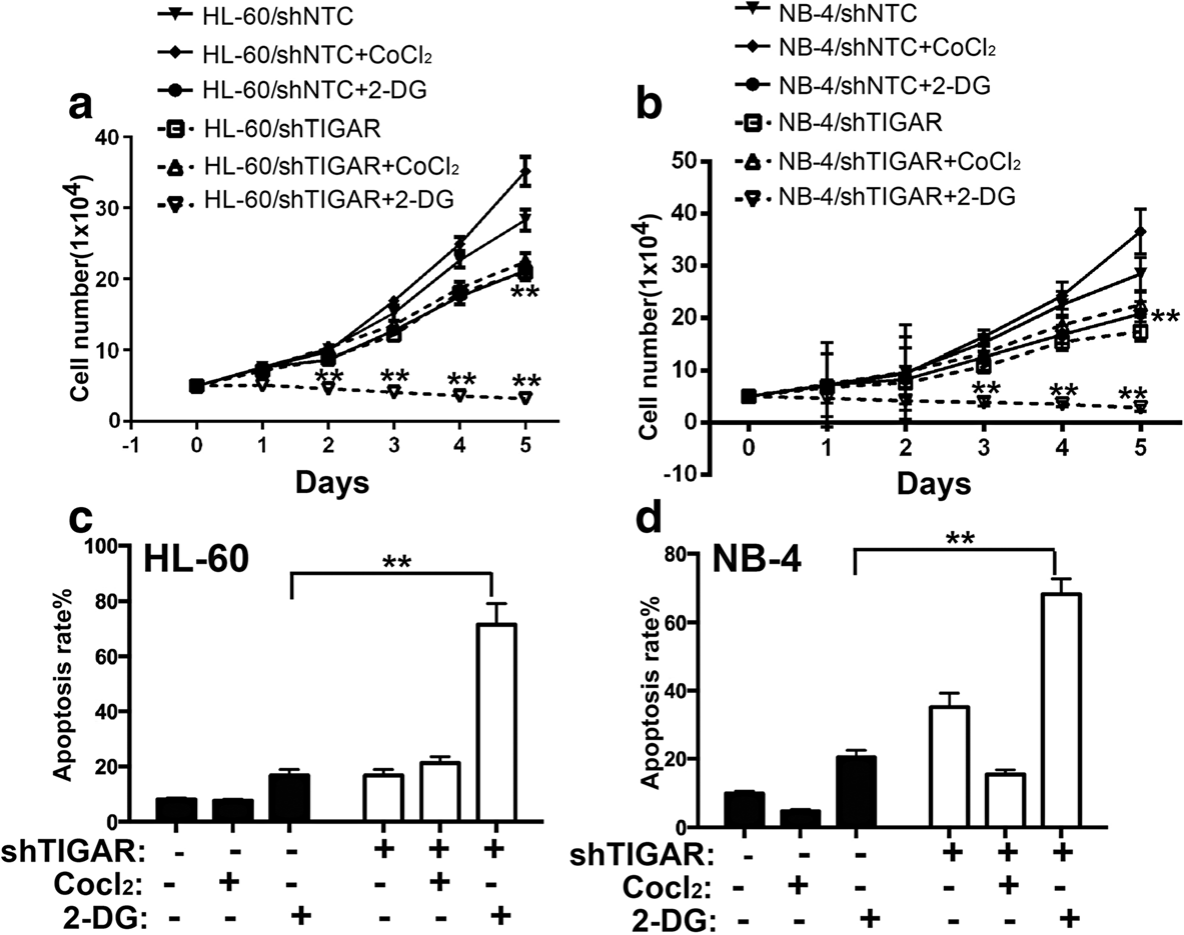
*TIGAR* knockdown inhibited the proliferation of leukemia cells and sensitized leukemia cells to glycolysis inhibition in vitro*.* **a** The cell proliferation assay showed the cell growth of HL-60 cells with *TIGAR* knockdown in combination with Cocl_2_ or 2-DG. **b** The cell proliferation assay showed the cell proliferation of NB-4 cells with *TIGAR* knockdown in combination with Cocl_2_ or 2-DG. **c**, **d** The cell apoptosis rate was determined by FACS in both HL-60 (**c**) and NB-4 cells (**d**) with *TIGAR* knockdown in combination with Cocl_2_ or 2-DG. HL-60 and NB-4 cells with or without *TIGAR* knockdown were treated with Cocl2 or 2-DG. The cells were collected on day 2 post Cocl2 or 2-DG treatment, and the apoptotic cells were determined by FACS

As TIGAR showed a high expression in primary AML cells and human acute leukemia cell lines, we next tested whether *TIGAR* knockdown may affect the proliferation of acute leukemia cells. Because *TIGAR* knockdown activated the glycolysis in leukemia cells, we also tested whether the glycolysis inhibitor may show a combinational effect with *TIGAR* knockdown. *TIGAR* shRNA constructs by targeting distinct *TIGAR* sequence was stably introduced into two different leukemia cell lines: HL-60 and NB-4. We next tested whether *TIGAR* knockdown affected the proliferation of HL-60 and NB-4 cells. Knockdown of *TIGAR* significantly inhibited the growth of both HL-60 and NB-4 cells (Fig. 4a, b). As we showed that glycolysis inhibitor 2-DG did not affect the expression of TIGAR, it suggested that 2-DG and TIGAR may affect the leukemia glycolysis through different mechanisms. Therefore, we tested whether *TIGAR* knockdown had a combinational effect with glycolysis inhibitor: 2-DG. *TIGAR* shRNA but not *NTC* shRNA showed a dramatically combination effect with 2-DG in both HL-60 and NB-4 cells (Fig. 4a, b). In contrast, *TIGAR* knockdown did not show any combinational effect with CoCl_2_ (Fig. 4a, b). Next, we determined to understand the potential mechanism of the combination effect of 2-DG and *TIGAR* knockdown. Under normal conditions, HL-60 and NB-4 cells showed a high TIGAR protein expression and a low level of apoptosis. Knockdown of *TIGAR* significantly increased leukemia cell apoptosis in both HL-60 and NB-4 cell lines (Fig. 4c), indicating a potential anti-apoptotic effect of TIGAR. CoCl_2_, inducting cell glycolysis, did not enhance the cell apoptosis in leukemia cells with or without *TIGAR* knockdown. However, 2-DG increased cell apoptosis in both leukemia cell lines. The combination of 2-DG and *TIGAR* knockdown significantly increased the leukemia cell apoptosis in comparison with either 2-DG or *TIGAR* knockdown (Fig. 4c). We also tested whether *TIGAR* knockdown led to the increase of cell death/necrosis in 2-DG treated HL-60 or NB-4 cells. We showed that *TIGAR* knockdown further enhanced the percentage of cell death/necrosis in 2-DG-treated leukemia cells (Additional file 3: Figure S3). These results suggested that *TIGAR* knockdown inhibited the proliferation of HL-60 and NB-4 cells, and 2-DG-caused glycolysis inhibition showed a synergistic effect with *TIGAR* knockdown in inhibiting leukemia cell proliferation.

### *TIGAR* knockdown sensitizes HL-60 leukemia cells to glycolysis inhibition in vivo

**Fig. 5:**
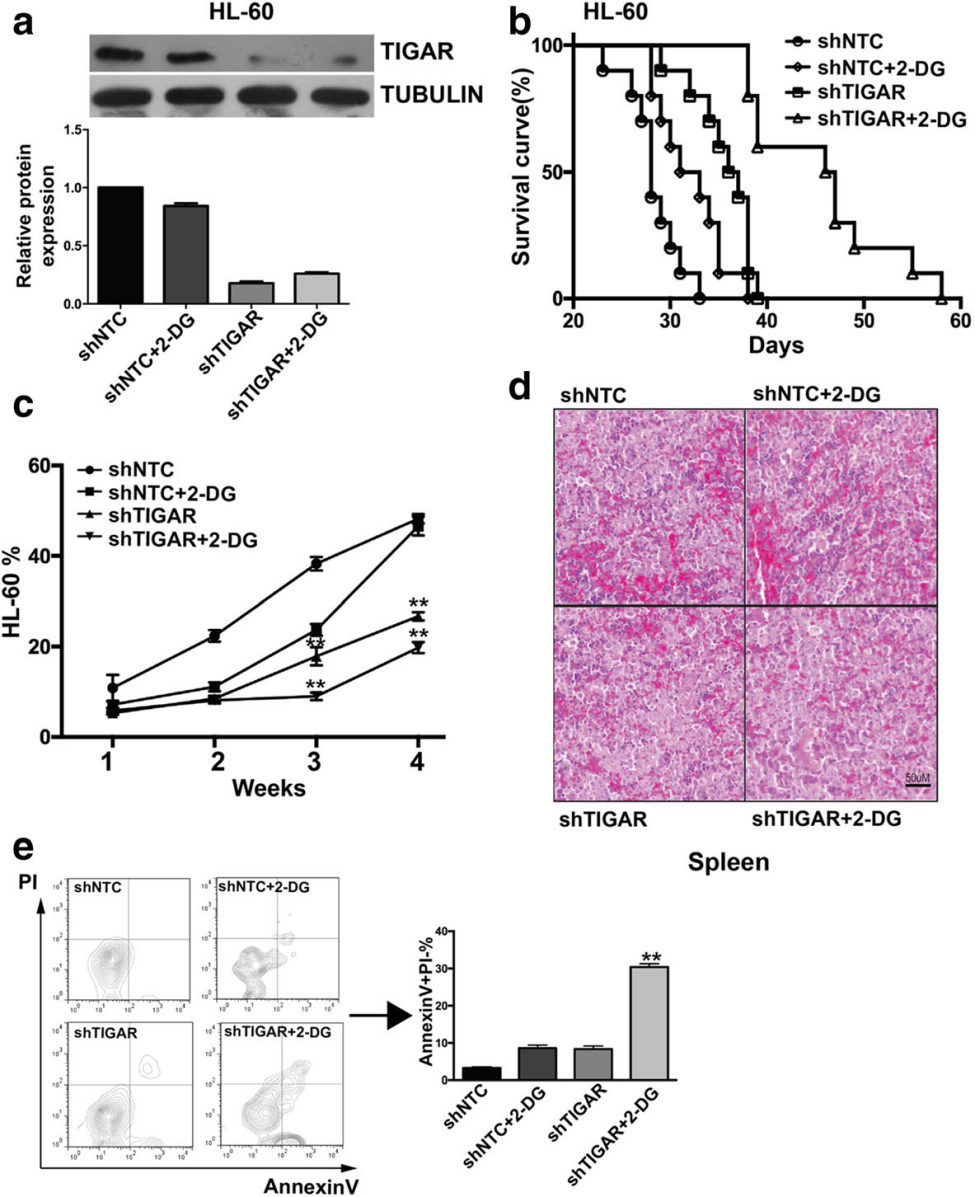
*TIGAR* knockdown sensitized HL-60 leukemia cells to glycolysis inhibition in vivo*.* **a** Western blotting analysis of HL-60 xenograft tumor samples. The ascites-derived tumor cells from mice were collected and lysed at the end of the study, and western blotting analyses of TIGAR and TUBULIN were performed (*n* = 2 for each group). **b** The survival of HL-60 xenograft mice with the combined treatment of *TIGAR* knockdown and 2-DG. 1 × 10^6^ HL-60 cells with or without *TIGAR* knockdown were inoculated into BALB/c (nu/nu) nude mice (*n* = 10). Those mice were treated or untreated with 2-DG (2 g/kg, PO, QD) from 1-week post implantation of HL-60 cells. **c** In HL-60 xenograft tumor mice, the effectiveness of *TIGAR* knockdown in combination with 2-DG in treating HL-60 xenograft tumor mice correlated with decreased percentages of HL-60 leukemia cells in PB. FACS analysis showed the decrease of HL-60 cells in PB of HL-60 xenograft tumor mice. Mean ± SD was shown. **d** Photomicrographs of hematoxylin and eosin-stained spleen sections from HL-60 xenograft tumor mice with the combined treatment of *TIGAR* knockdown and 2-DG. **e** The combined treatment of *TIGAR* knockdown and 2-DG induced apoptosis of HL-60 leukemia cells in mice. The HL-60 cells from ascites fluid were collected and stained with PI and Annexin-V, and the percentages of PI^−^/Annexin-V^+^, representing apoptotic cells, were determined by FACS (*n* = 5). Mean ± SD was shown

To further validate the effect of *TIGAR* knockdown on leukemia cell proliferation, the effect of *TIGAR* knockdown and *TIGAR* knockdown in combination with 2-DG were tested in HL-60 xenograft mouse model. *TIGAR* shRNA alone inhibited HL-60 cells growth by around 45%, and the survival of AML mice was extended, and knockdown was confirmed (Fig. 5a–c). 2-DG alone at tolerated dosage (10 mg/kg PO, qd) did not inhibit HL-60 leukemia cell growth at the endpoint but extended survival of AML mice mildly (Fig. 5a–c). More strikingly, *TIGAR* knockdown and 2-DG combination inhibited HL-60 cells growth by 59% and extended the survival of HL-60 cells xenograft mice significantly (Fig. 5a–c). *TIGAR* knockdown and 2-DG combination also significantly reduced leukemia cells in the spleens from HL-60 cells xenograft mice (Fig. 5d). We also measured the apoptosis rates among different groups. As expected, the combination of 2-DG and *TIGAR* knockdown significantly increased the leukemia cell apoptosis (Fig. 5e). These results suggest that TIGAR is important for glycolysis of leukemia cells, and *TIGAR* knockdown sensitizes human leukemia cells to glycolysis inhibition both in vitro and in vivo.

### The expression and functional effect of TIGAR were uncoupled from p53 in HL-60 and NB-4 cells

**Fig. 6:**
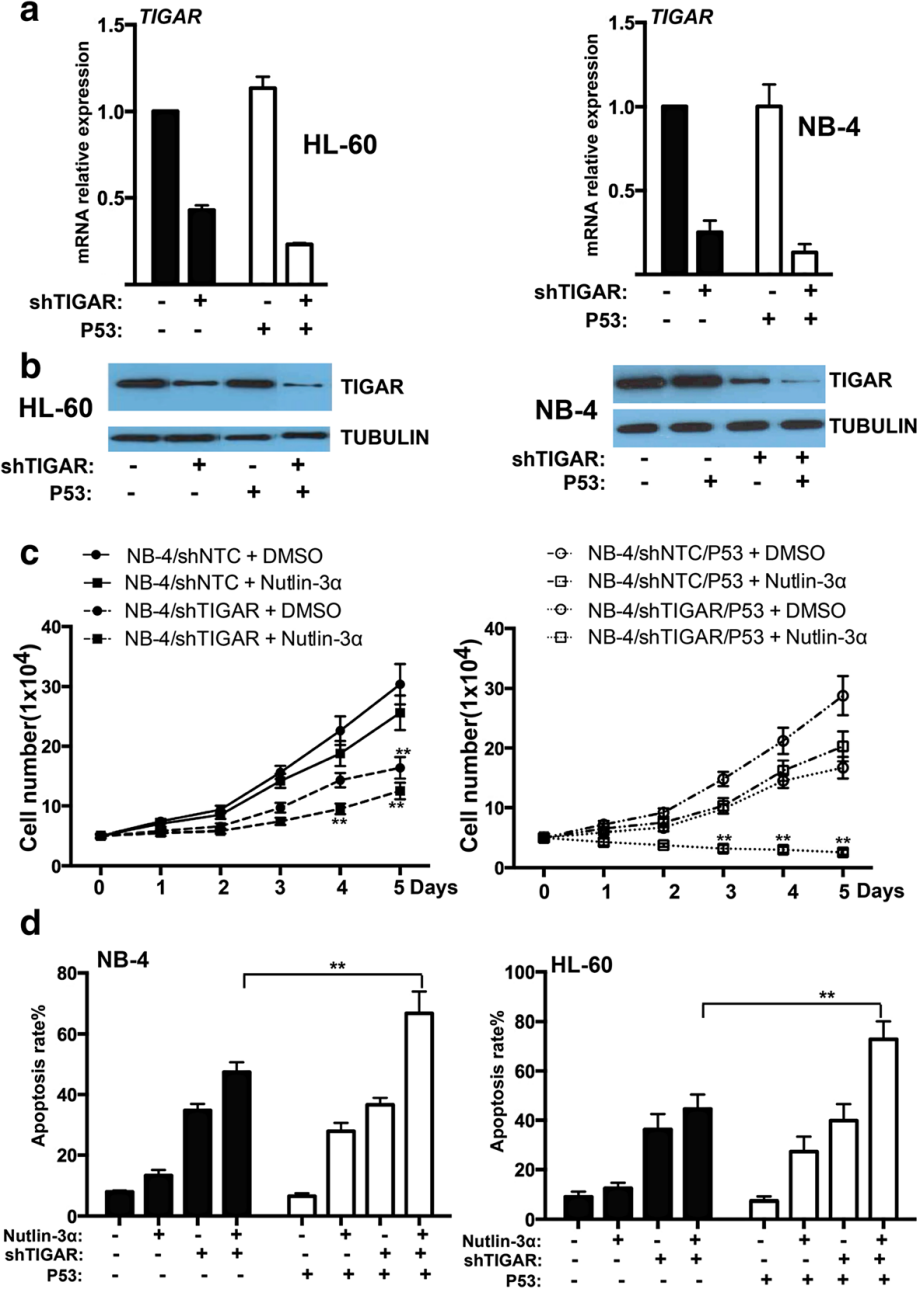
TIGAR expression and its anti-apoptotic effect were uncoupled from p53 in human leukemia cells. **a** Real-time PCR showed that the mRNA expression of *TIGAR* was not affected by p53 overexpression in both HL-60 and NB-4 cells. **b** Western blotting showed that the protein expression of TIGAR was not affected by p53 overexpression in both HL-60 and NB-4 cells. **c** The cell proliferation assay showed the cell growth of NB-4 cells with *TIGAR* knockdown in combination with p53 overexpression or/and MDM2 inhibitor Nutlin-3α. **d** *TIGAR* knockdown in combination with p53 overexpression or/and MDM2 inhibitor Nutlin-3α induced the apoptosis of NB-4 and HL-60 cells in vitro

p53 is disabled in HL-60 and NB-4 cell lines by either deletion (HL-60) or missense mutation (NB-4) of the p53 gene. As TIGAR was induced by p53 and protected cancer cell from death, we next investigated whether the expression or function of TIGAR may be affected by overexpression p53 in leukemia cells. We stably transfected with wild type p53 into leukemia cells and the overexpression of p53 was confirmed by western blot (Additional file 4: Figure S4). We found that TIGAR expression was mildly enhanced by p53 in both HL-60 and NB-4 cells stably transfected with p53 (Fig. 6a, b). In addition, *TIGAR* shRNA showed a better knockdown effect on leukemia cells transfected with p53. Those results implied that TIGAR expression might be uncoupled from p53 in leukemia cells.

We also examined whether overexpression of p53 may affect the proliferation of human leukemia cells. Overexpression of p53 did not affect the proliferation of NB-4 cells. In contrast, MDM2 inhibitor Nutlin-3α is shown to induce p53-mediated apoptosis [26] and showed a significant combinational effect with *TIGAR* knockdown in NB-4 leukemia cells. *TIGAR* knockdown/Nutlin-3α/p53 overexpression showed a best effect on inhibiting leukemia cell proliferation (Fig. 6c). Consistent with leukemia cell proliferation, leukemia cell apoptosis was robustly increased by the combination of p53 overexpression, *TIGAR* knockdown, and Nutlin-3α in both HL-60 and NB-4 leukemia cells (Fig. 6d). In addition, the inhibition of cell proliferation may be due to cell death/necrosis. We also measured the cell death/necrosis and showed that the cell death/necrosis rate was relatively low among the different groups (Additional file 5: Figure S5). These results suggested that overexpression of p53 only slightly affected TIGAR expression in human leukemia cells, and p53 activation had a combinational effect on inhibiting leukemia cell proliferation and promoting leukemia cell apoptosis.

## Discussion

Intracellular processes drived multiple hallmarks of cancer have highlighted the potential to affect oncogenesis and cancer progression by manipulating these critical processes at a molecular level [27]. In our study, the prognostic relevance of TIGAR expression in patients with CN-AML, suggested that higher TIGAR expression might be an independent poor prognostic factor, irrespective of age, WBC count, karyotype, and other genetic markers. Chemotherapy regimen was also an important factor to affect the outcome of AML patients. In our study, three chemotherapy regimens were used among AML patients with high or low TIGAR expression. The multivariate analysis showed that these chemotherapy regimens (*P* = 0.078) as well as BM blast (*P* = 0.058) may also affect the outcome of patients with CN-AML. Furthermore, high expression of TIGAR showed an anti-apoptotic effect on human leukemia cells, which may contribute to the poor OS and higher cumulative incidence of relapse in patients with CN-AML treated with chemotherapy. The relationship between TIGAR expression and prognosis in patients with solid cancers was also shown in multiple studies [11, 15, 28]. Similar with human acute leukemia cells, the increased expression of TIGAR was able to protect against metabolic stress, contributes to tumor growth, and be uncoupled from its normal dependence on p53 in several cancer cell types [4]. A number of genetic alterations seen in CN-AML patients with possible prognostic relevance (DNMT3A, IDH1/2, TET2) are not considered here, and the correlation of TIGAR expression and outcome may not be independent of other variables in CN-AML. In addition, *TIGAR* knockdown enhanced the radiosensitivity of cancer cells, suggesting that correlation of TIGAR expression and outcome of patients with CN-AML may also depend on the response of AML cells to chemotherapy [13]. In the future, it will be important to understand how TIGAR affects the response of leukemia cells to chemotherapy or targeted therapy.

**Fig. 7:**
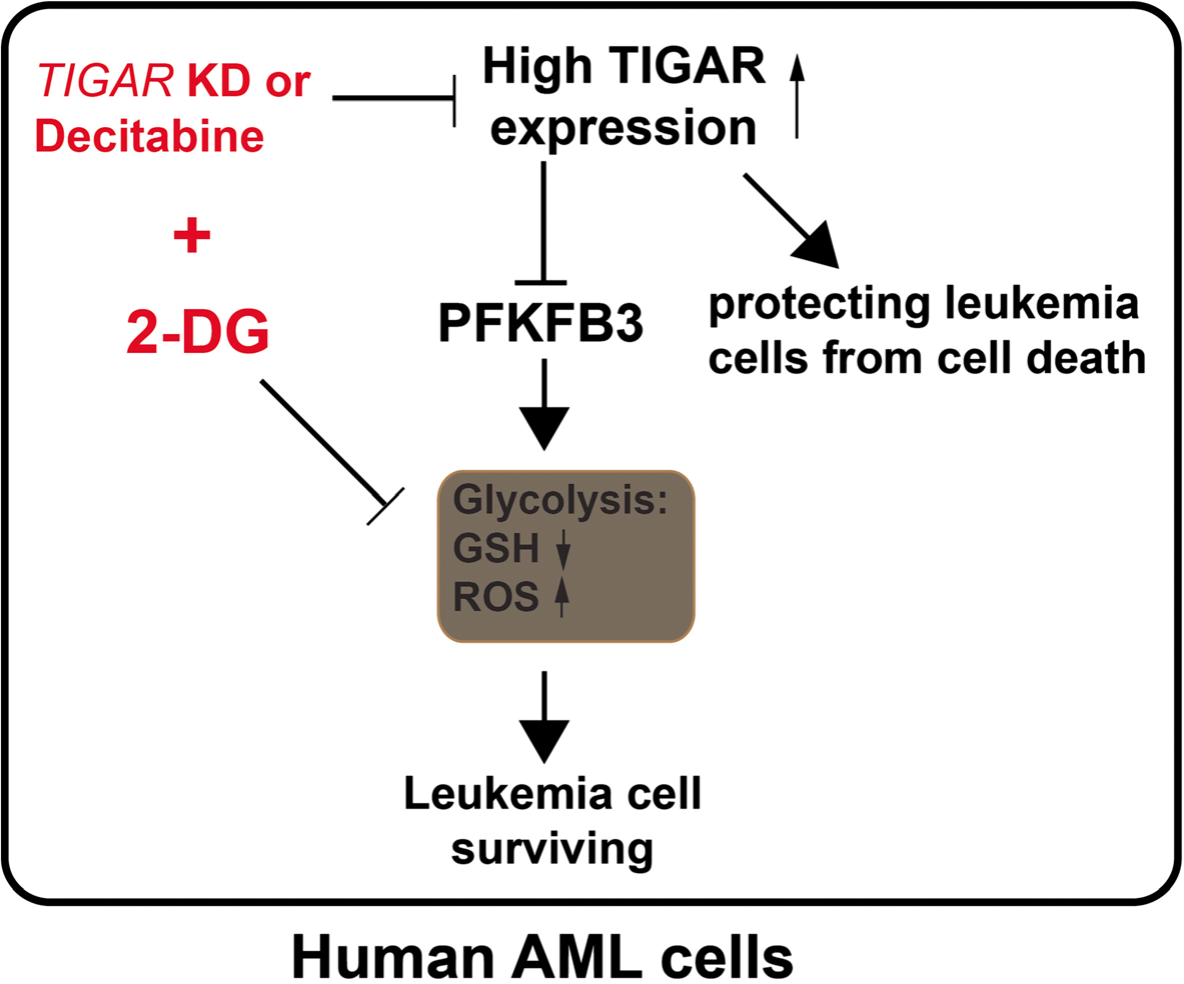
A working model of TIGAR regulating the glycolysis and the proliferation of human leukemia cells. TIGAR played a dual role in human AML cells. It protected the leukemia cells from cell death while inhibited PFKFB3-regulated glycolysis in AML. Thus, *TIGAR* knockdown or decitabine induced apoptosis of leukemia cells through inhibiting the expression of TIGAR and sensitized leukemia cells to glycolysis inhibition in human AML

TIGAR might have a dual effect on the proliferation of leukemia cell (Fig. 7). On the one hand, TIGAR inhibited glycolysis through PFKFB3 in leukemia cells [29]. TIGAR inhibited glycolysis, decreased ROS, and increased GSH levels. Knockdown of *TIGAR* induced while overexpression of *TIGAR* reduced the expression of glycolysis activator PFKB3 in leukemia cells. High levels of glycolysis in cancer cells have been linked to poor prognosis and chemotherapy resistance [30, 31]. Highly glycolytic AML blasts are more resistant to combined all-trans retinoic acid and arsenic trioxide treatment than moderately glycolytic blasts [31]. Therefore, high expression of *TIGAR* inhibited the glycolysis of leukemia cells and led leukemia cells to sensitive to chemotherapy. On the other hand, our results also showed that TIGAR protected leukemia cells from cell death. Knockdown of *TIGAR* led a significant increase of cell apoptosis in human leukemia cells. In contrast, *TIGAR* knockdown also promoted the glycolysis in leukemia cells. Our studies showed that TIGAR related anti-apoptosis and lower glycolysis might maintain a steady-state. Our results also showed that glycolysis inhibition or *TIGAR* knockdown alone only caused the limited apoptosis of leukemia cells. In contrast, a robust leukemia cell apoptosis was observed when leukemia cells with simultaneous impairment of *TIGAR* expression and glycolysis. In addition, *TIGAR* knockdown in combined with 2-DG also mildly enhanced cell death/necrosis of leukemia cells. The mechanism of TIGAR regulating the cell apoptosis or necrosis and glycolysis of leukemia cells was still not clear. More studies should be done to illustrate the underlying process.

TIGAR might be a novel target for treating human acute leukemia. Knockdown of *TIGAR* inhibited the proliferation of human leukemia cells and sensitized leukemia cells to glycolysis inhibitor 2-DG both in vitro and in vivo*.* In addition, *TIGAR* knockdown in combination with 2-DG led leukemia cells to apoptosis. As suppressing glycolysis with inhibitors was not effective at killing tumor cells, its combination with other tumor-specific metabolic inhibitors may be necessary for therapeutic intervention [32]. Silencing *TIGAR* also enhanced the radiosensitivity of U87MC and glioma cells [13]. Our findings suggest that combining glycolytic inhibitors with a potential TIGAR inhibitor and current standard chemotherapy may be a powerful and effective treatment for not only human leukemia but also other cancer types. More efforts are needed to develop the potent and specific small molecule compound for TIGAR.

However, TIGAR expression and its anti-apoptotic effect were uncoupled from p53 in human leukemia cells. In normal cells, *TIGAR* transcription was rapidly activated by p53 in response to low levels of cellular stress. In human tumor cells, the expression of TIGAR was regulated by p53 dependent or p53-independent mechanisms [8]. In wild-type p53 tumor cell line, the basal expression of TIGAR was relatively low and the induction of p53 led to an increased expression of TIGAR protein [8]. However, p53 null or mutant human tumor cell lines including H1299, U2OS, and RKO showed a significant basal level of TIGAR protein expression, which indicated the existence of p53-independent mechanisms to regulate TIGAR expression in human cancer cells [8]. In human p53 null or mutant leukemia cells, overexpression of p53 did not significantly induce the expression of TIGAR. Our observation was also supported by other studies, which also showed that *TIGAR* expression was regulated by other non-p53 mechanisms in human cancer cell line [8, 33, 34].

## Conclusions

In summary, our study showed that high *TIGAR* expression was associated with poor survival and a high incidence of relapse in adult patients with CN-AML, even after adjustment for known clinical and common molecular risk factors. Moreover, sustained TIGAR activation, uncoupled from p53, may support AML cell growth and survival. TIGAR in cooperation with glycolysis had a strong anti-apoptotic effect in AML cells. Therefore, the combination of TIGAR inhibitors with anti-glycolytic agents may be powerful novel therapies for the future clinical use in AML patients.

## Additional files

Additional file 1: Figure S1. F2,6BP level was affected by Cocl_2_ or 2-DG in HL-60 cells. (JPEG 860 kb)
Additional file 2: Figure S2. a. PFKFB3 regulated glycolysis in cancer cells. b. Western blotting showed the expression of PFKFB3 in HL-60 cells with *TIGAR* knockdown and in K562 cells with *TIGAR* overexpression. c. Western blotting showed the expression of TIGAR and PFKFB3 in HL-60 cells treated with decitabine in combination with Cocl_2_ or 2-DG. d. Western blotting showed the expression of TIGAR and PFKFB3 in NB-4 cells treated with Cocl_2_ or 2-DG. (JPEG 1536 kb)
Additional file 3: Figure S3. The cell death/necrosis rate was determined by FACS in both 2-DG treated HL-60 and NB-4 cells with or without *TIGAR* knockdown. (JPEG 851 kb)
Additional file 4: Figure S4. Overexpression of p53 in NB-4 or HL-60 cells. (JPEG 1341 kb)
Additional file 5: Figure S5.
*TIGAR* knockdown in combination with p53 overexpression or/and MDM2 inhibitor Nutlin-3α did not affect the cell death/necrosis of NB-4 cells in vitro*.* NB-4 cells with or without TIGAR knockdown in combination with p53 overexpression or/and MDM2 inhibitor Nutlin-3α were collected on day 2 post Nutlin-3α treatment, and the death cells were determined by FACS. (JPEG 943 kb)

## Abbreviations

2-DG: 2-Deoxy-d-glucose
AML: Acute myeloid leukemia
CN-: Cytogenetically normal
CoCl2: Cobalt chloride
CR: Complete remission
DFS: Disease-free survival
FBPase-2: Bisphosphatase domain
Fru-2: 6-P2, fructose-2, 6-bisphosphate
GSH: Glutathione
OS: Overall survival
PFK-2/FBPase: 6-phosphofructo-2-kinase
PI: Propidium iodide
PPP: Pentose phosphate pathway
ROS: Reactive oxygen species
WBC: White blood cell count
WT: Wild-type
