# Supplementary figures and images for: TIGAR cooperated with glycolysis to inhibit the apoptosis of leukemia cells and associated with poor prognosis in patients with cytogenetically normal acute myeloid leukemia
Source: J Hematol Oncol. 2016 Nov 25;9:128. doi: 10.1186/s13045-016-0360-4 (PMC5123356; doi:10.1186/s13045-016-0360-4)

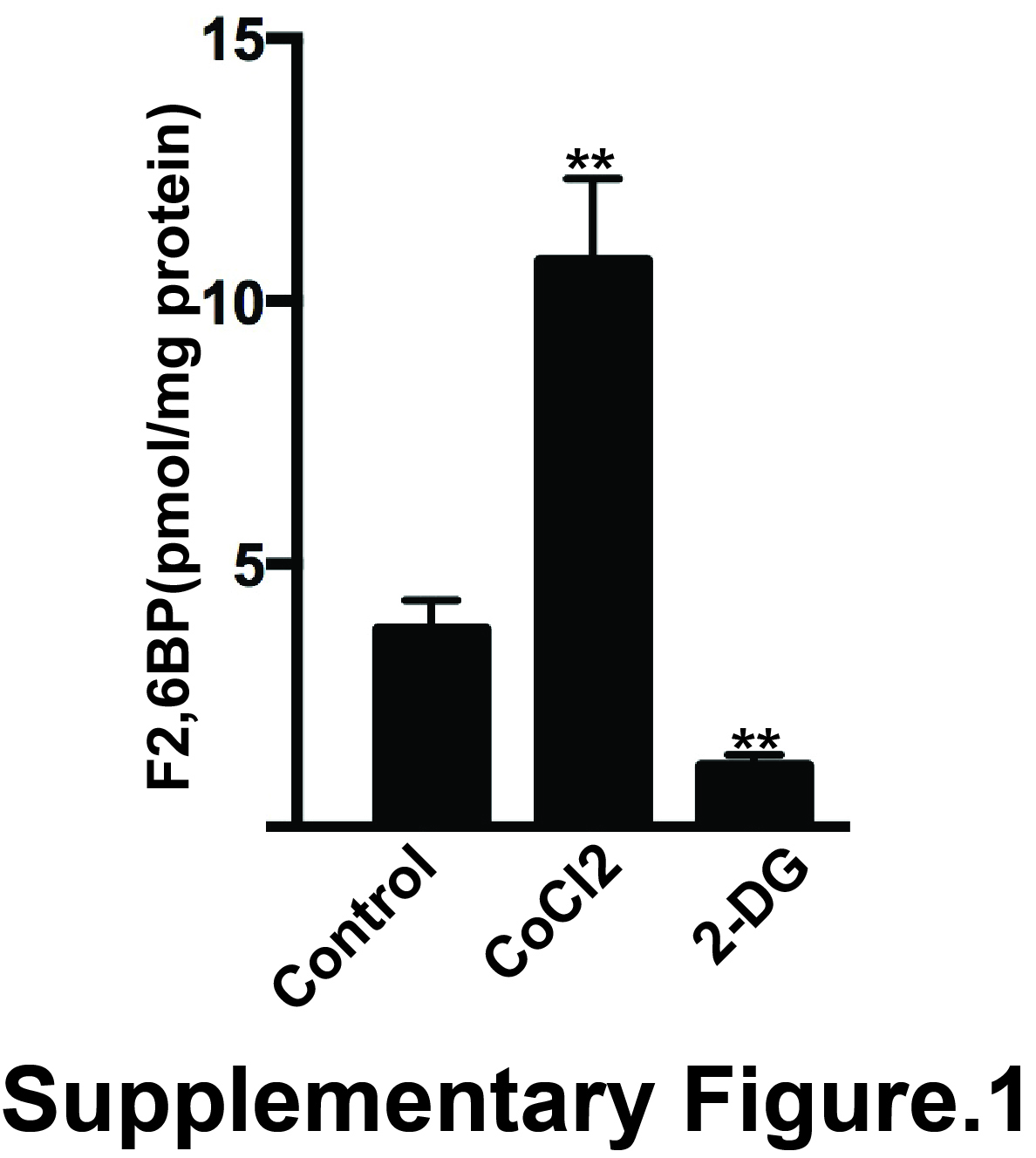

Supplement: Additional file 1: Figure S1. — F2,6BP level was affected by Cocl2 or 2-DG in HL-60 cells. (JPEG 860 kb) [file 13045_2016_360_MOESM1_ESM.jpg]

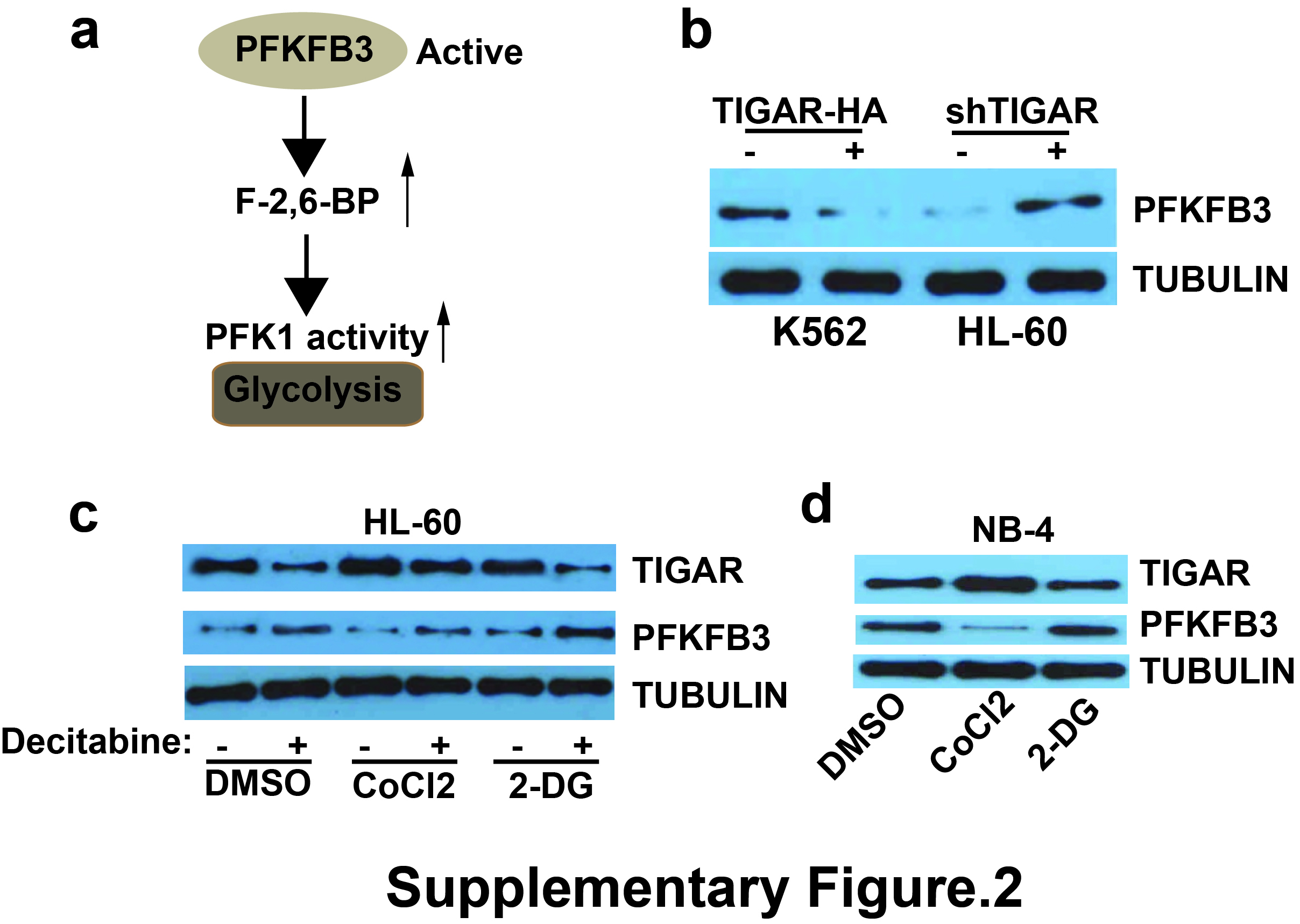

Supplement: Additional file 2: Figure S2. — a. PFKFB3 regulated glycolysis in cancer cells. b. Western blotting showed the expression of PFKFB3 in HL-60 cells with TIGAR knockdown and in K562 cells with TIGAR overexpression. c. Western blotting showed the expression of TIGAR and PFKFB3 in HL-60 cells treated with decitabine in combination with Cocl2 or 2-DG. d. Western blotting showed the expression of TIGAR and PFKFB3 in NB-4 cells treated with Cocl2 or 2-DG. (JPEG 1536 kb) [file 13045_2016_360_MOESM2_ESM.jpg]

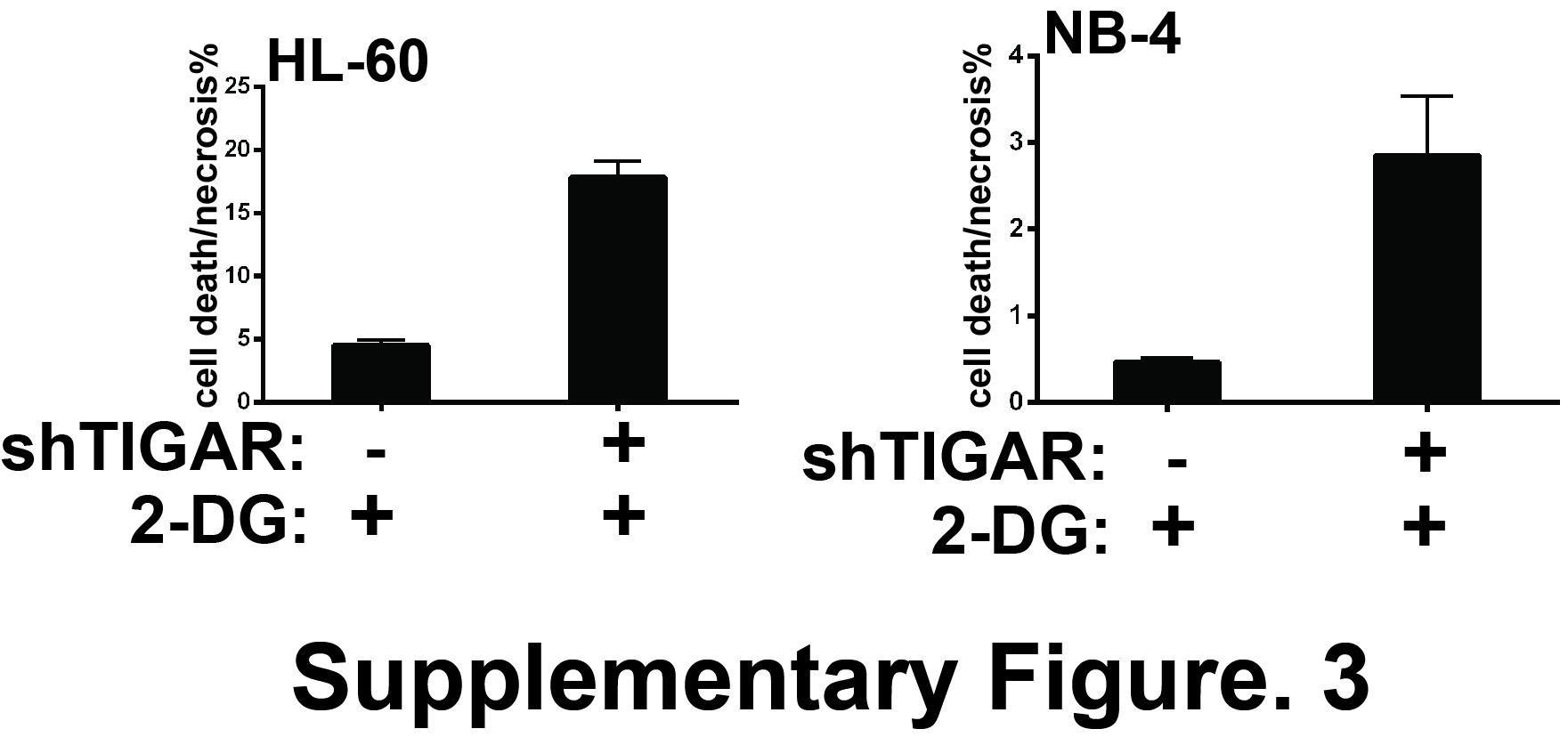

Supplement: Additional file 3: Figure S3. — The cell death/necrosis rate was determined by FACS in both 2-DG treated HL-60 and NB-4 cells with or without TIGAR knockdown. (JPEG 851 kb) [file 13045_2016_360_MOESM3_ESM.jpg]

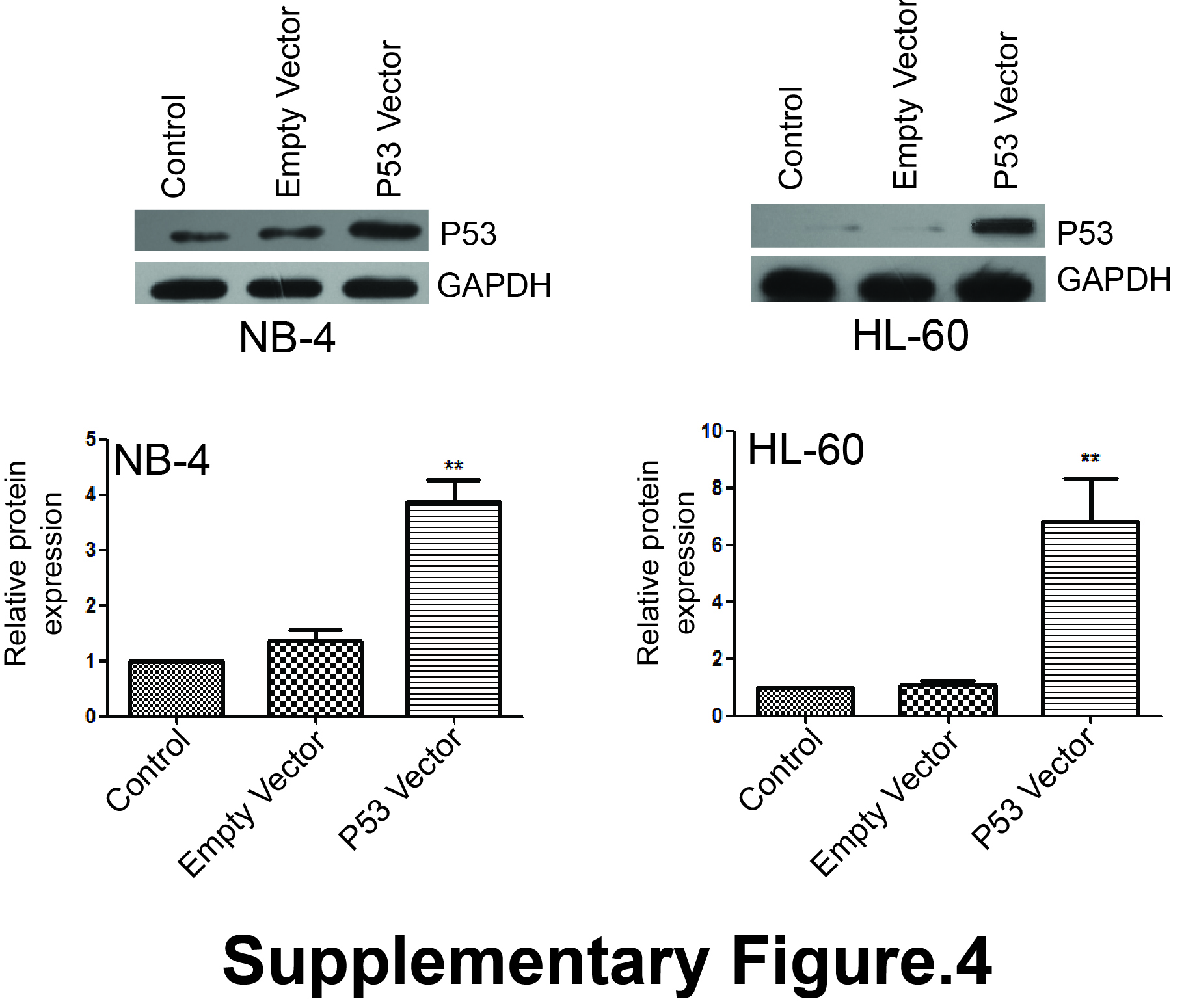

Supplement: Additional file 4: Figure S4. — Overexpression of p53 in NB-4 or HL-60 cells. (JPEG 1341 kb) [file 13045_2016_360_MOESM4_ESM.jpg]

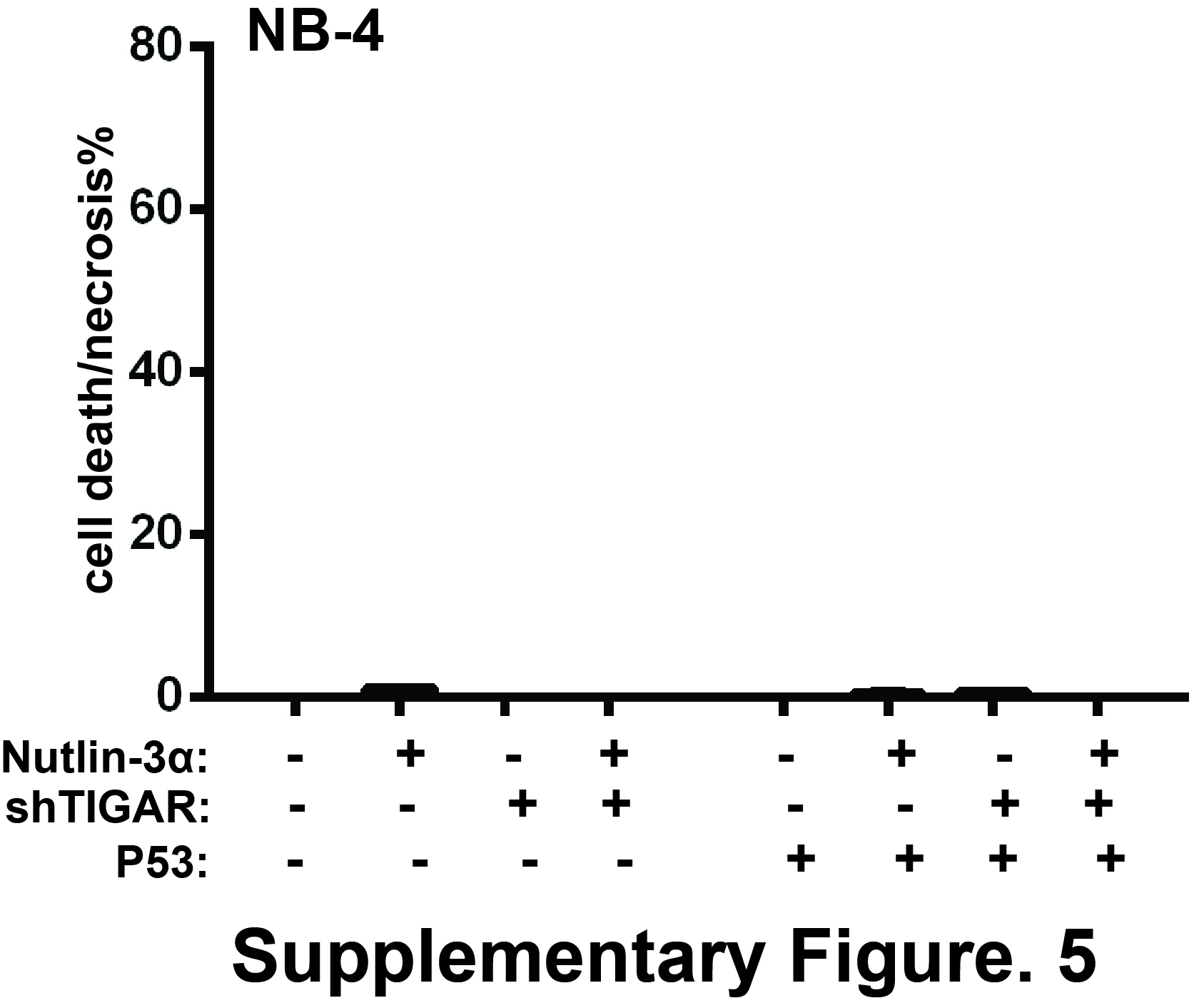

Supplement: Additional file 5: Figure S5. — TIGAR knockdown in combination with p53 overexpression or/and MDM2 inhibitor Nutlin-3α did not affect the cell death/necrosis of NB-4 cells in vitro. NB-4 cells with or without TIGAR knockdown in combination with p53 overexpression or/and MDM2 inhibitor Nutlin-3α were collected on day 2 post Nutlin-3α treatment, and the death cells were determined by FACS. (JPEG 943 kb) [file 13045_2016_360_MOESM5_ESM.jpg]
